# Right Coronary Artery-to-Right Atrial Fistula Accompanied by Multiple Right Coronary Artery Aneurysms: A Case Report

**DOI:** 10.7759/cureus.56398

**Published:** 2024-03-18

**Authors:** Kevin Watat, Georgette Nader, Rand Sabanci, Adolfo Martinez, Manel Boumegouas, Christopher A Hanson, Matthew Wilcox

**Affiliations:** 1 Internal Medicine, Michigan State University, East Lansing, USA; 2 Internal Medicine, BronxCare Health System, New York, USA; 3 Department of Cardiology, Sparrow Hospital Thoracic and Cardiovascular Institute, Lansing, USA

**Keywords:** arteriovenous fistula, coronary computed tomoangiography, coronary aneurysm, coronary arterial fistula, right coronary artery (rca)

## Abstract

A coronary artery aneurysm (CAA) denotes a localized dilation of the coronary artery, while a coronary artery fistula signifies an aberrant connection between a coronary artery and a cardiac chamber or adjacent vessel. Here, we present a case study of a 68-year-old female with a previously diagnosed right coronary artery-to-right atrial fistula concomitant with multiple right coronary artery aneurysms. Initially asymptomatic, the patient subsequently manifested atrial fibrillation. Management involved augmenting the patient’s home regimen with metoprolol tartrate, followed by successful cardioversion and restoration of sinus rhythm. Given the stability of the fistula and the absence of symptomatic exacerbation, no further interventional measures were undertaken. The patient was discharged with an adjusted metoprolol regimen and scheduled follow-up with her cardiologist. Subsequent imaging assessments unveiled progressive fistula expansion alongside the development of concurrent CAA, inciting deliberations concerning optimal treatment modalities.

## Introduction

A coronary artery fistula (CAF) constitutes a relatively uncommon vascular anomaly, which may manifest as either congenital or acquired in etiology, characterized by the establishment of an abnormal vascular connection between a coronary artery and either a cardiac chamber or neighboring vasculature [1]. Although typically encountered as an isolated entity, certain instances demonstrate an association with adjacent coronary artery aneurysms (CAAs), suggesting a potential interplay between these pathological conditions [2].

CAA represents a rare vascular phenomenon, with an incidence ranging from 0.3% to 4.9%, defined by localized arterial dilation exceeding 1.5 times the diameter of adjacent normal vessels, with “giant” CAA denoted by diameters exceeding 2 cm [1,3]. The right coronary artery (RCA) emerges as a frequent site of involvement in both CAA and CAF occurrences, highlighting the anatomical predilection and potential pathophysiological correlation within the coronary vasculature. Additionally, the right atrium serves as a common site for vascular drainage in these cases [4].

Clinical manifestations associated with these vascular anomalies vary widely, ranging from asymptomatic presentations to manifestations of potentially life-threatening complications, thereby necessitating a comprehensive approach to diagnosis and management. The concurrent presentation of both CAF and CAA represents an exceedingly rare event, signifying the diagnostic challenges and therapeutic complexities encountered in clinical practice [3]. Hence, the determination of treatment necessity is contingent upon multiple factors. In this context, we present a detailed case report involving a 68-year-old individual presenting with a confirmed right coronary artery-to-right atrial (RCA-RA) fistula concomitant with multiple right coronary artery aneurysms (RCAAs), elucidating the clinical intricacies and therapeutic considerations in managing this complex vascular pathology.

This article was previously presented as a poster at the 2023 American College of Cardiology Annual Scientific Meeting on March 4, 2023.

## Case presentation

**Figure 1:**
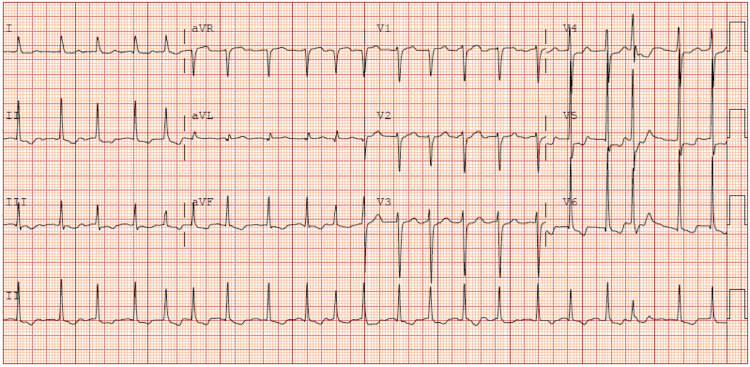
Electrocardiogram demonstrating atrial fibrillation with RVR (HR: 127 beats/minute) with premature ventricular complex and left ventricular hypertrophy. Image obtained from the patient’s chart. RVR: rapid ventricular rate; HR: heart rate

**Figure 2:**
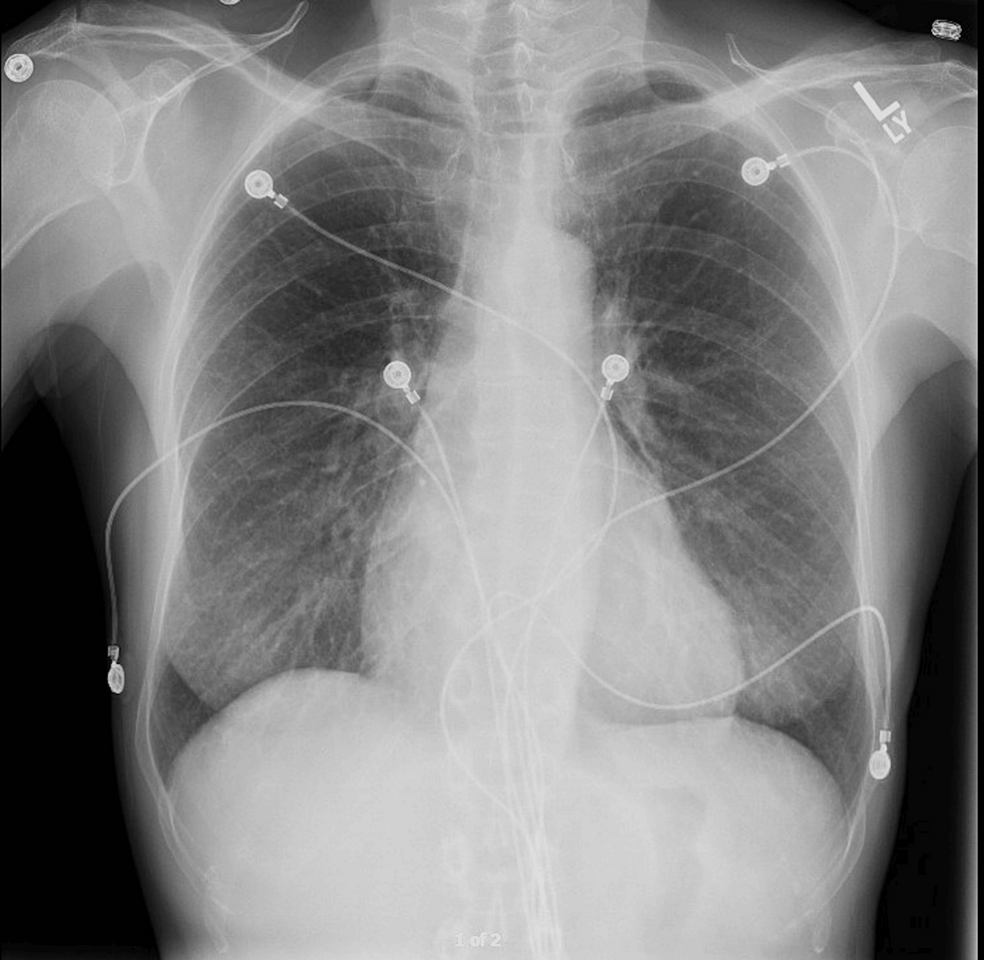
Chest X-ray on admission showing mild cardiomegaly. Image obtained from the patient’s chart.

A 68-year-old female with a medical history notable for hypertension and hyperthyroidism presented to an external emergency department with a recent onset of dizziness and palpitations over the course of several days. Upon clinical examination, she exhibited tachycardia alongside normal respiratory rate and blood pressure parameters. Initial laboratory investigations revealed elevated levels of troponin-I at 0.14 and hypokalemia (Table 1). Electrocardiographic (assessment unveiled the emergence of new-onset atrial fibrillation accompanied by a rapid ventricular rate, premature ventricular complexes, and signs indicative of left ventricular hypertrophy (Figure 1). Radiographic examination via chest X-ray disclosed mild cardiomegaly devoid of intrathoracic pathology (Figure 2). The patient was administered aspirin and subsequently transferred to the primary hospital facility for further comprehensive cardiological evaluation.

**Table 1:** Initial workup on admission.

Investigation	Values	Reference range
Blood pressure	126/61 mmHg	120/80 mmHg
Heart rate	114 beats/minute	60–90 beats/minute
Respiration rate	18 breaths/minute	12–20 breaths /minute
Initial troponin-I	0.14 ng/mL	0–0.03 ng/mL
Repeat troponin-I	0.10 ng/mL	0–0.03 ng/mL
Potassium	3.4 mEq/L	3.5–4.9 mEq/L
Serum bicarbonate	25.8 mmol/L	20–32 mmol/L
Anion gap	11	2–16
Aspartate transaminase	34 U/L	10–40 U/L
Alanine transaminase	21 U/L	2–45 U/L
Bilirubin	1.3 mg/dL	0.2–1.2 mg/dL
Blood urea nitrogen	19 mg/dL	6–23 mg/dL
Creatinine	0.9 mg/dL	0.6–1.4 mg/dL
White blood cell count	8.8 × 10^3^/µL	4-12 × 10^3^/µL
Hemoglobin	13.6 g/dL	12.6–16.5 g/dL
Platelet count	237 × 10^3^/µL	150–400 × 10^3^/µL

Significantly, the patient was identified as having a sizable arteriovenous fistula (AVF) originating from the RCA-RA, with a left-to-right shunt ratio of 1.36:1, which was visualized during cardiac catheterization conducted as part of her dyspnea assessment in 1992. Subsequently, in 2003, she presented to the cardiology clinic with complaints of intermittent dyspnea and palpitations, prompting a reassessment of the fistula via left and right heart catheterization. This evaluation revealed a stable AVF, albeit with a modest increase in saturation observed from the superior vena cava to the pulmonary artery. Notably, her symptoms spontaneously resolved following this evaluation and she remained asymptomatic for 13 years.

Upon admission to the primary hospital, the patient’s treatment regimen was initiated with a heparin infusion, concomitant with an escalation of her metoprolol tartrate dosage from 50 mg daily to 100 mg daily. Subsequent evaluation via transesophageal echocardiogram (TEE) revealed normal left ventricular systolic function, a negative result on the agitated saline bubble study, absence of thrombus within the left atrial appendage, and persistence of a stable 2.4 cm RCA-RA fistula. Successful cardioversion to normal sinus rhythm was achieved via a single 150 J shock, resulting in the resolution of her symptoms. Given the patient’s asymptomatic status and maintenance of appropriate functional capacity, the decision was made to abstain from surgical intervention for the RCA-RA fistula. Consequently, the heparin infusion was ceased, and treatment was transitioned to apixaban at a dosage of 5 mg twice daily. The patient’s condition remained stable, facilitating discharge on the subsequent day with scheduled follow-up appointments at the outpatient cardiology clinic.

**Figure 3:**
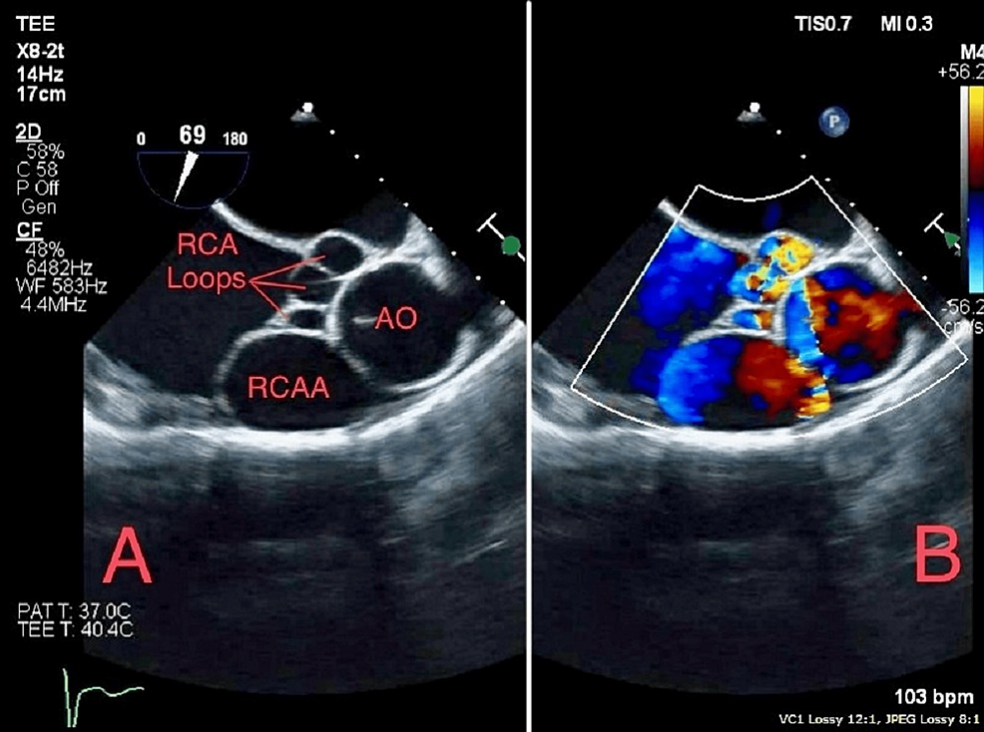
(A) Transesophageal echocardiogram demonstrating RCAA and RCA-RA fistula. (B) Blood flow is represented. Image obtained from the patient’s chart. RCAA: right coronary artery aneurysm; AO: aorta; RCA: right coronary artery

**Figure 4:**
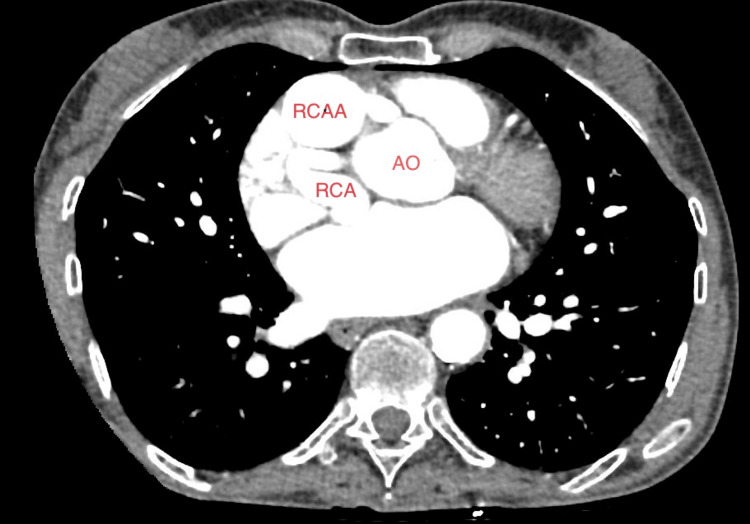
Coronary CTA demonstrating proximal RCAA. Image obtained from the patient’s chart. CTA: computed tomographic angiography; RCAA: right coronary artery aneurysm; RCA: right coronary artery

**Figure 5:**
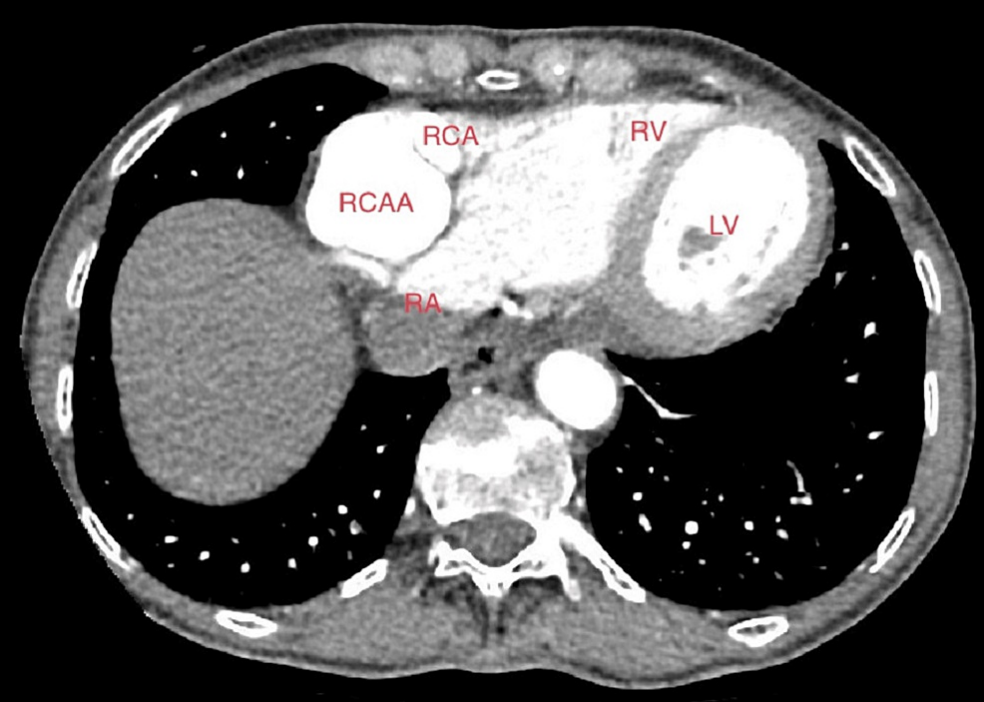
Coronary CTA demonstrating distal RCAA. Image obtained from the patient’s chart. CTA: computed tomographic angiography; RCAA: right coronary artery aneurysm; RCA: right coronary artery; RV: right ventricle; LV: left ventricle; RA: right atrium

Subsequent follow-up TEE unveiled a progressive enlargement of the AVF alongside the emergence of a novel RCAA (Figures 3A, 3B). The most recent documented size of the RCA-RA fistula measured 2.9 × 2.9 cm, whereas the latest coronary computed tomography angiography (CTA) showcased proximal and distal RCAA dimensions measuring 3.7 × 3.4 × 4.7 cm and 4.4 × 4.4 × 3.5 cm (Figures 4, 5). Notably, a point of anastomosis was identified between the distal RCA and the inferior portion of the superior vena cava (ISVC). This anatomical configuration could potentially represent an RCA-ISVC fistula.

## Discussion

CAA represents a noteworthy pathological entity characterized by focal dilation exceeding 1.5 times the diameter of the adjacent normal coronary artery [1]. With an incidence ranging from 0.3% to 4.9%, CAA typically arises in the native coronary artery proximate to atheromatous lesions and may coincide with the presence of CAFs [3]. The latter, albeit even rarer, demonstrates an incidence of 0.1% to 0.2%, with a notable predilection for the RCA [3]. In fact, RCA involvement constitutes approximately 33% of documented cases, with the right ventricle (34%) and right atrium (27%) being the most commonly observed drainage sites [4].

The pathogenesis underlying both CAA and CAF remains subject to debate, although their etiologies are believed to encompass congenital and acquired factors. CAA predominantly arises from atherosclerotic processes in adults; however, it can also manifest in the context of congenital heart defects, Kawasaki disease, vasculitides (such as Takayasu arteritis and polyarteritis nodosa), as well as certain autoimmune disorders (e.g., scleroderma and systemic lupus erythematosus) [5]. Conversely, CAF primarily stems from traumatic events (such as gunshot wounds or stab injuries) or iatrogenically through interventional cardiac procedures [6]. Clinical presentations span a spectrum from asymptomatic cases to acute coronary syndromes, ruptures, and arrhythmias [3]. The majority of CAA/CAF instances are asymptomatic and are incidentally detected via imaging modalities. Definitive diagnosis necessitates direct visualization through coronary angiography, regarded as the gold standard diagnostic tool.

Symptomatology appears to correlate with the magnitude of the shunt or the presence of coronary steal phenomenon at the level of the fistula [3,7]. Substantial shunt volumes may induce or exacerbate the enlargement of aneurysms within the supplying coronary artery, thereby impeding the functional integrity of the recipient cardiac chamber and leading to compromised myocardial oxygenation [4,7]. This mechanism provides a plausible explanation for the gradual enlargement observed in both the RCAA and the RCA-RA fistula in our patient, particularly in light of the documented left-to-right shunt identified on imaging since 1992. This progressive enlargement likely contributed to the onset of atrial fibrillation. Although rare, previous literature reports have documented cases of CAF presenting concomitantly with atrial fibrillation [4,8]. In line with our case, visualization of the RCA-RA fistula on imaging suggests a comparable pathophysiological mechanism. Consequently, it is plausible to infer that the anatomical location of the fistula likely precipitated the development of atrial fibrillation in this instance.

Management strategies for CAF remain contentious, hinging upon the presence or absence of symptoms, which are, in turn, contingent upon the severity of shunting [1,4]. Timely intervention, either through surgical or percutaneous repair/closure, is advocated to forestall complications across both symptomatic and asymptomatic cohorts. However, the prevailing literature predominantly recommends surgical or percutaneous intervention for symptomatic individuals, while advocating vigilant monitoring for asymptomatic patients [1,3]. Nonetheless, asymptomatic patients deemed to be at elevated risk necessitate intervention. Risk stratification hinges on factors such as the course, tortuosity, and concomitant aneurysmal dilation of the fistula [4]. Percutaneous closure holds preference over surgical approaches owing to its minimally invasive nature [1,4].

The classification of our patient into the appropriate management category was subject to debate. Although presenting with atrial fibrillation, this manifestation could have been attributable to hyperthyroidism rather than directly linked to the fistula, rendering her asymptomatic from a fistula perspective. Nevertheless, given the coexistence of an aneurysm, she could be deemed high risk. Ultimately, our decision to opt for monitoring was predicated upon the stable imaging findings of the RCA-RA fistula and RCAA over successive evaluations, coupled with the patient’s notably swift recovery post-cardioversion.

## Conclusions

CAFs and CAAs represent exceedingly rare pathological entities, with their concomitant occurrence being even more infrequent. Notably, these anomalies exhibit a predilection for the RCA, with the RCA-RA and RCA-RV fistulas being particularly prevalent. Diagnosis typically occurs incidentally during imaging investigations. The clinical spectrum of these conditions is broad, encompassing various presentations, among which arrhythmias, including atrial fibrillation, can serve as initial manifestations.

Recommendations advocate for surgical or percutaneous intervention for both symptomatic and asymptomatic patients. Nonetheless, asymptomatic individuals managed conservatively have demonstrated limited complication risks, underscoring the potential efficacy of medical management alone.
